# Triterpenoids-templated self-assembly nanosystem for biomimetic delivery of CRISPR/Cas9 based on the synergy of TLR-2 and ICB to enhance HCC immunotherapy

**DOI:** 10.1016/j.apsb.2024.04.033

**Published:** 2024-05-08

**Authors:** Bing-Chen Zhang, Chun-Mei Lai, Bang-Yue Luo, Jing-Wei Shao

**Affiliations:** a Fujian Provincial Key Laboratory of Cancer Metastasis Chemoprevention and Chemotherapy, College of Chemistry, Fuzhou University, Fuzhou 350116, China; b Department of Laboratory Medicine, Dongguan Institute of Clinical Cancer Research, the Tenth Affiliated Hospital of Southern Medical University (Dongguan People's Hospital), Dongguan 523058, China; c College of Materials and Chemical Engineering, MinjiangUniversity, Fuzhou, 350108, China

**Keywords:** Ursolic acid, Self-assembly, Biomimetic nanoplatform, Hepatocellular carcinoma, CRISPR/Cas9, Immune checkpoint blockade, Gene therapy, Immunotherapy

## Abstract

Combination immunotherapy has shown promising potential for enhancing the objective response rate compared to immune checkpoint blockade (ICB) monotherapy. However, combination therapy with multi-drugs is limited by the different properties of the agents and inconsistent synergistic targeted delivery. Herein, based on a universal triterpene template and the anticancer active agent ursolic acid (UA), a cytomembrane-coated biomimetic delivery nanoplatform (UR@M) prepared by the self-assembly of a *PD-L1* targeted CRISPR/Cas9 system and UA was designed for hepatocellular carcinoma (HCC) treatment. UR@M showed enhanced tumor accumulation *in vivo* with homologous tumor targeting, and CRISPR in the nanosystem exhibited potent gene-editing efficiency of 76.53% *in vitro* and 62.42% *in vivo* with no off-target effects. UA activated the natural immune system through the TLR-2-MyD88-TRAF6 pathway, which synergistically enhanced the proliferation of natural killer cells and dendritic cells and realized excellent immune cytotoxic T cell infiltration by combining with the ICB of *PD-L1*. The strategy of work along both lines based on innate immune and adaptive immunity displayed a significant effect in tumor regression. Overall, the UA-templated strategy “killed three birds with one stone” by establishing a self-assembly nanosystem, inducing tumor cell death, and promoting synergistic immunostimulation for HCC treatment.

## Graphical abstract

A biomimetic nanodrug UR@M from the triterpenoids-templated self-assembly is developed to deliver CRISPR/Cas9, exhibiting synergistic immunotherapy by activating innate immune by TLR-2 pathway and gene therapy of PD-L1 by CRISPR/Cas9.

**Figure:**
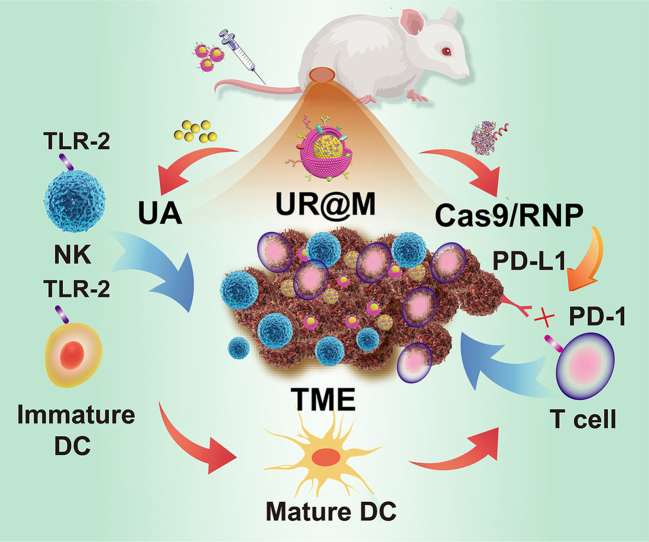

## 1 Introduction

Immunotherapy has become an important strategy in clinical cancer treatment and has attracted much attention, especially for the immune checkpoint blockade (ICB) of *PD-1/PD-L1* as a clinically validated treatment for manifold-type terminal cancers^1^^,^^2^. Recent research has shown the limitations of the systemic administration of ICB therapy using antibodies or small-molecule drugs, including low target specifically, risk of an autoimmune response, and limitations due to large individual differences^3^^,^^4^. With the advent of CRISPR/Cas9 gene-editing technology^5^, the research progress of tumor ICB gene therapy has been greatly accelerated^6^^,^^7^. Gene therapy directly acts on immune checkpoint genes with higher gene editing efficiency and therapeutic effects, the destruction of immune checkpoint gene locus and downregulation of PD-1/PD-L1 expression by CRISPR/Cas9 technology effectively reduce the proliferation and spread of cancer cells^8^^,^^9^. However, the immune response rate is low in patients with terminal hepatocellular carcinoma (HCC), which is also confirmed to be in the state of the immunosuppressor tumor microenvironment (ITM)^10^, ^11^, ^12^. Compared to ICB monotherapy, combination therapy is a novel clinical strategy for treating HCC^13^^,^^14^.

With the deepening of research on the active ingredients of natural products, some flavonoids, polysaccharides, and terpenoids derived from medicinal plants have been used as adjuvant treatments with salutary pharmacological activity to promote treatment and reduce side effects^15^, ^16^, ^17^. Ursolic acid (UA) is a pentacyclic triterpenoid extracted from natural products with biological functions, such as anticancer, antibacterial, and anti-metastasis activity^18^, ^19^, ^20^. Our previous studies showed that UA not only exhibited an effective inhibitory effect on tumor cell proliferation but also demonstrated a unique immune-activating effect^21^^,^^22^. The pluralistic immune activation through innate and adaptive immunity effectively overcame the tumor ITM, indicating a potential combined therapeutic strategy to enhance ICB therapy. Considering the potential biosafety of viral vectors^23^, the novel nanotechnology has been proved an available delivery system for the application of CRISPR/Cas9 in cancer treatment^24^^,^^25^. However, the low drug loading and unknown metabolism of nanocarriers *in vivo* remain potent obstacles to clinical application^26^^,^^27^. Notably, we found that UA was a universal self-assembly template that could be used to combine a variety of different hydrophobic or hydrophilic molecules to form an integrated nanodrug^28^, ^29^, ^30^, ^31^, ^32^. Based on the specific “deliver drug by drug” system, the UA-template had the greater advantage by constructing self-assembled nanoparticles with simple preparation, high drug loading, low toxicity side effects and slow degradation^33^, ^34^. In addition, with the help of the mature biomimetic system, the homologous tumor cell membrane further enhanced the targeted delivery of drugs and reduced the immune phagocytosis of drugs *in vivo*^35^, ^36^, ^37^. Due to the highly polymerized structure, self-assembly nanoparticles allowed cell membranes to be coated around the surface layers, instead of adding the PLGA or other framework molecule in the preparation of a biomimetic system based on the cell membrane in previous research^38^.

**Scheme 1:**
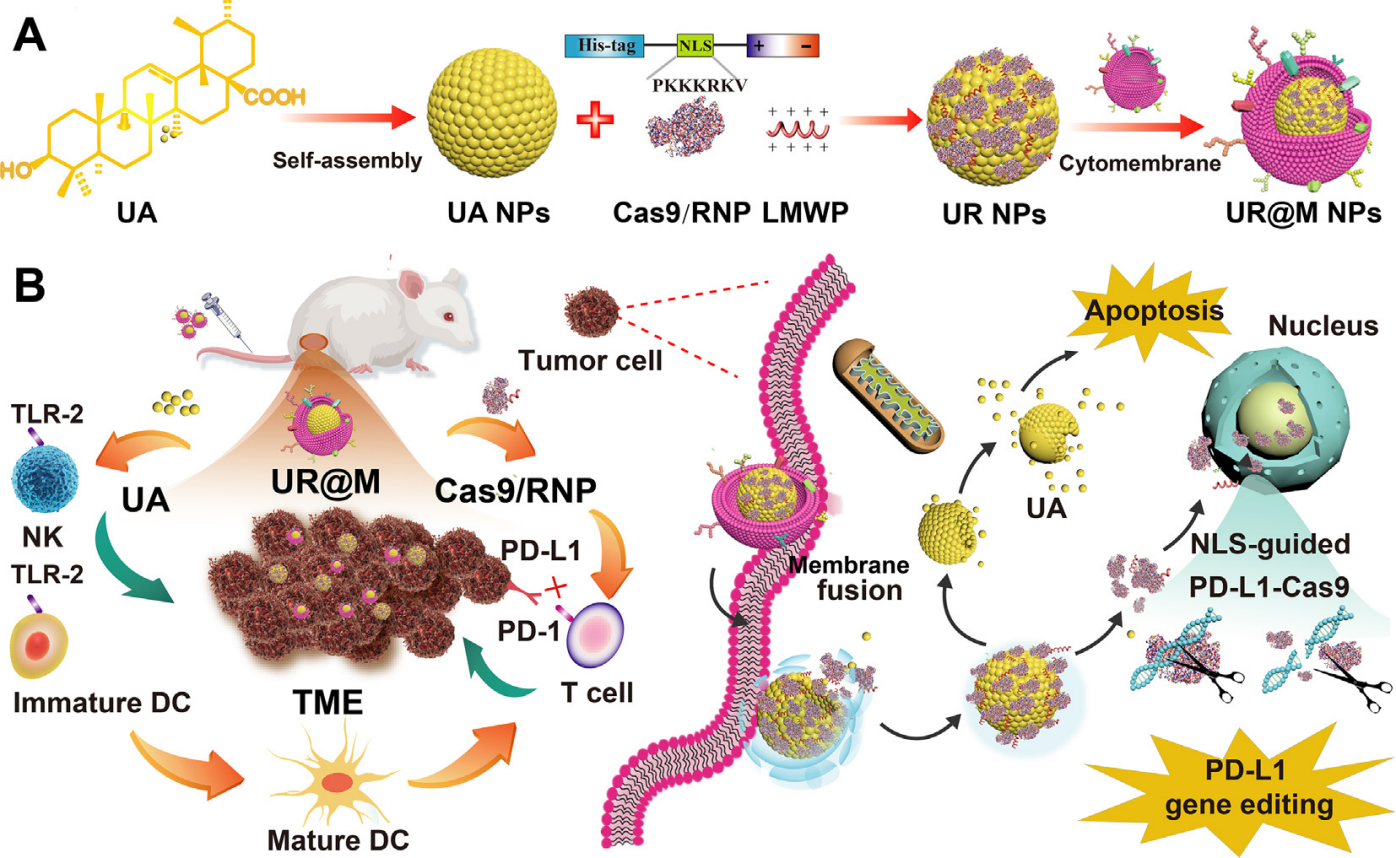
Schematic illustration of therapeutic mechanism of UR@M NPs for HCC immunotherapy. (A) Schematic design of the biomimetic UR@M NPs based on the UA and CRISPR system. (B) Schematic illustration of nanodrug for enhanced immunotherapy by the combination of UA and gene therapy of PD-L1.

In the current study, a *PD-L1*-targeted CRISPR/Cas9 system was first designed for use in gene editing by the Cas9/sg-*PD-L1* ribonucleoprotein complex (RNP). Secondly, a self-assembly nanodrug of UR formed by UA, Cas9/RNP, and a penetrating peptide (low-molecular-weight protamine, LMWP) was prepared. LMWP was introduced to increase the charge balance and stability of the system, as well as to improve tumor cell uptake by the transmembrane effect. Finally, a biomimetic delivery system (UR@M NPs) was constructed by coating HCC cytomembranes on the surface of UR (Scheme 1A). By the homologous targeting of cell membranes and the rapid penetration of self-assembled NPs, UR@M could enhance cancer cell uptake and drug accumulation in the tumor. In addition to the anti-cancer properties, the efficient immune activation and reversal of ITM by UA was evaluated, and it was suggested to be an available combination strategy to assist the ICB gene therapy of *PD-L1* (Scheme 1B). Moreover, the HCC therapeutic effect and biological safety evaluation were also examined in animal models.

## 2 Materials and methods

### 2.1 Materials

Ursolic acid (UA) and indocyanine green (ICG) were bought from Macklin (Macklin, U820363/I953656, Shanghai, China). Serum for cell culture was purchased from Inner Mongolia Opcel Biotechnology Co., Ltd. (OPCEL, BS-1101, Huhehaote, China). The crRNAs of PD-L1 (Supporting Information Table S1) we designed were synthesized and provided by Sangon Biotech Co., Ltd. (Sangon, Shanghai, China).

### 2.2 Preparation of PD-L1 gene targeted CRISPR/Cas9 system

The Cas9 protein was expressed by *E. coli* BL21 cells, which were transformed with pET-Cas9-NLS-6xHis plasmids. After the protein was purified and concentrated, the content was quantified by micro-ultraviolet spectrophotometry. Afterward, *PD-L1*-targeted crRNAs and tracrRNA were synthesized by Integrated DNA Technologies. *PD-L1*-sgRNA was constructed by slowly annealing from 95 to 20 °C. Finally, Cas9 protein and sgRNA were incubated in distilled deionized water at molar ratios of 1:2 for 30 min at 37 °C to generate the *PD-L1*-targeted Cas9/RNP complex.

### 2.3 Preparation of UR@M

First, 4 mg of UA dissolved in 1 mL of methanol was slowly added to 9 mL of deionized water under stirring at 1000 rpm (Eppendorf 5424R, Hamburg, Germany). Methanol was removed by nitrogen blowing. Then, 200 μg of Cas9/RNP and 67 μg of LMWP were mixed in 1 mL of deionized water, slowly poured into the UA NP solution, and stirred at 1000 rpm (Eppendorf) for 10 min. The free small molecules and proteins were filtered through dialysis bags (MWCO: 2000) to obtain self-assembled UR NPs. HepG2 cells were collected and added to hypotonic phosphate-buffered saline (PBS) with 1% phenylmethyl sulfonyl fluoride (PMSF) for 30 min. Cell impurities were first removed by low-speed centrifugation (4 °C, 500×*g*, 10 min, Eppendorf), and then the supernatant was performed a further high speed centrifugation (4 °C, 14,000×*g*, 30 min, Eppendorf) to obtain the cytomembrane, which was freeze-dried and stored at −80 °C. Finally, 1 mL of cytomembrane solution (1 mg/mL) was mixed with 9 mL of UR NP solution under ultrasonication for 15 min, and the biomimetic nanodrug (UR@M) was prepared by a couple of extrusions through a 200-nm polycarbonate membrane.

### 2.4 Characterization of UR@M

The particle size and zeta potential of nanodrugs were measured on a Mastersizer. The morphologies were evaluated using transmission electron microscopy (TEM, FEI, Tecnai G2F20, Hillsboro, Oregon, USA) and atomic force microscopy (AFM, Nano Surface Division, Bruker, USA). The integrity of cell membrane proteins on the surface of the nanodrug was detected by polyacrylamide gel. The stability of the nanodrugs was investigated by the continuous monitoring of particle size. The drug release of UR was investigated in PBS and serum. One milliliter of UR solution was poured into the dialysis bag (MWCO: 5000), which was placed in 9 mL of PBS or serum. PBS was selected to simulate the different pH environments. During the stirring process of solutions, the samples were taken at the indicated time points and detected. The concentration of UA was analyzed by HPLC and protein was detected by ultramicro-spectrophotometry.

### 2.5 Cellular uptake of UR@M

The clinically approved contrast agent of indocyanine green (ICG) was used to evaluate the cellular uptake of the nanodrug, and the free ICG was regarded as the control. HepG2 cells were incubated in a 6-well plate for 24 h. Then, the drug of different groups (free ICG, UR, UR@M) with the same concentration of UA and ICG was added. The concentration of free ICG was the same as that of ICG in nanodrugs (20 μmol/L). At the indicated time points, the cells were washed twice and the 500 μL of PBS containing Hoechst 33342 was added for 10 min. The cells were treated with cell fixation solution. Finally, the fluorescence imaging of tumor cells was captured by confocal laser scanning microscope (CLSM, Zeiss, LSM 900, Oberkochen, Germany). Nuclear and drugs were detected by fluorescence channels at 450 and 639 nm, respectively.

### 2.6 Cytotoxicity assay

The cytotoxicity of nanodrugs was investigated by the co-culture of human leukemia Jurkat cells and tumor cells by CCK-8 assay. Experimental groups treated with UA, UA NPs, Cas9/RNP∗, Cas9/RNP, UR and UR@M were set up. First, the Jurkat (1 × 10^4^ cells/well) and tumor cells (1 × 10^4^ cells/well) were incubated for 24 h. Then, the 100 μL of fresh medium containing various concentrations of the indicated drugs (UA: 1, 5, 10, 15, 20, and 30 μg/mL; Cas9/RNP: 0.05, 0.25, 0.5, 0.75, 1, 1.5 μg/mL) was added for 24 h. CCK8 solution was added after washing by PBS and the removal of suspended Jurkat cells. The cytotoxicity of the nanodrugs was evaluated based on the absorbance.

### 2.7 Evaluation of gene editing efficiency

T7E1 enzymatic digestion was performed to evaluate the gene-editing efficiency of the nanodrug. PBS and UA groups were regarded as negative controls. UR and UR@M nanodrugs were added to the cells for 6 h, after which the cells were cultured for 48 h. Then, genomic DNA was extracted to obtain the targeted gene segment by polymerase chain reaction (PCR). Finally, the T7E1 enzyme was added to the reaction system for enzymatic digestion. The newly generated DNA fragment was used to investigate gene therapy efficiency by ImageJ.

### 2.8 *In vivo* tumor suppression

Kunming mice (KM, 4–5 weeks old) were provided by Guangdong Medical Laboratory Center. All animals used in the research were handled following protocol procedures, approved by the Institutional Animal Care and Use Committee of Southern Medical University. The xenograft tumor models were prepared by subcutaneously injecting H22 cells (1 × 10^6^ cells in 100 μL of 0.9% NaCl solution) after the cells were activated in the flank of the mouse. The drug concentration of UA (6 mg/kg) and Cas9/RNP (0.2 mg/kg) was injected in each mouse once every two days. The volumes of the tumors were calculated during the treatment periods. After the treatment on Day 21, the tumors were excised and prepared for immunofluorescent staining and TUNEL assay. The major organs and tumors were performed for H&E staining. The serum was collected to detect the blood biochemical index. The orthotopic tumor model was established by the hepatic injection of H22 tumor cells (3 × 10^6^ cells in 100 μL of 0.9% NaCl solution) with surgery. The liver was collected after the treatment of 21 days to investigate the therapeutic efficiency of nanodrugs.

### 2.9 *In vivo* fluorescence imaging

To track the distribution of nanodrugs in HCC tumor-bearing mice, *in vivo* fluorescence imaging was captured by IVIS Spectrum. ICG was also used for the *in vivo* imaging. With the free ICG as the control, UR NPs and UR@M NPs labeled with ICG (20 μmol/L) were intravenously injected into mice, and the fluorescence images were captured within 24 h. After the *in vivo* fluorescence imaging, mice were euthanized and the excised organs were detected by IVIS Spectrum. In the process of the experiment, fluorescence intensity quantification of different of organs was recorded.

### 2.10 Expression of genes in innate immunity pathway

At first, peripheral blood mononuclear cell (PBMCs) of tumor-bearing mice was obtained from the whole blood. The UA, Cas9/RNP, UR, UR@M were added into the PBMCs for 24 h, and then the mRNA and protein were extracted to evaluate the expression of the relative gene. The qRT-PCR primers used to quantify *TLR-2*, *TLR-4*, *TLR-6*, *TLR-3*, *cGAS*, and *RIG-1* were listed in Table S1. Then, the protein was quantified by a BCA kit, and the proteins were electrophoresed with SDS-PAGE. After transmembrane and protein closure, the primary antibody and fluorescent secondary antibody was used for WB, and the protein expression of TLR-2, MyD88, and TRAF6 was characterized by the imaging system of Odyssey.

### 2.11 Synergistic immune-activated effects of UR@M

The blood and tumor were collected after the treatment. The frequency of CD4^+^/CD8^+^ T and NK cells was examined by flow cytometry with the fluorescent antibody. Meanwhile, the serum was extracted from the blood, and the concentration of related cytokines in the serum was examined by the corresponding ELISA kit. To examine the immune infiltration of CD8^+^ T cells, the single-cell suspension of tumors was marked with the specific fluorescent antibody, and it was investigated by flow cytometric measurement.

### 2.12 Statistical analysis

All those experiments were performed at least three times, the results were expressed as mean ± SD. In addition, the *t*-test, and chi-squared test were used for significance analysis. ∗*P* < 0.05, ∗∗*P* < 0.01, ∗∗∗*P* < 0.001 were considered significant.

## 3 Results and discussion

### 3.1 Preparation and characterization of UR@M

**Figure 1:**
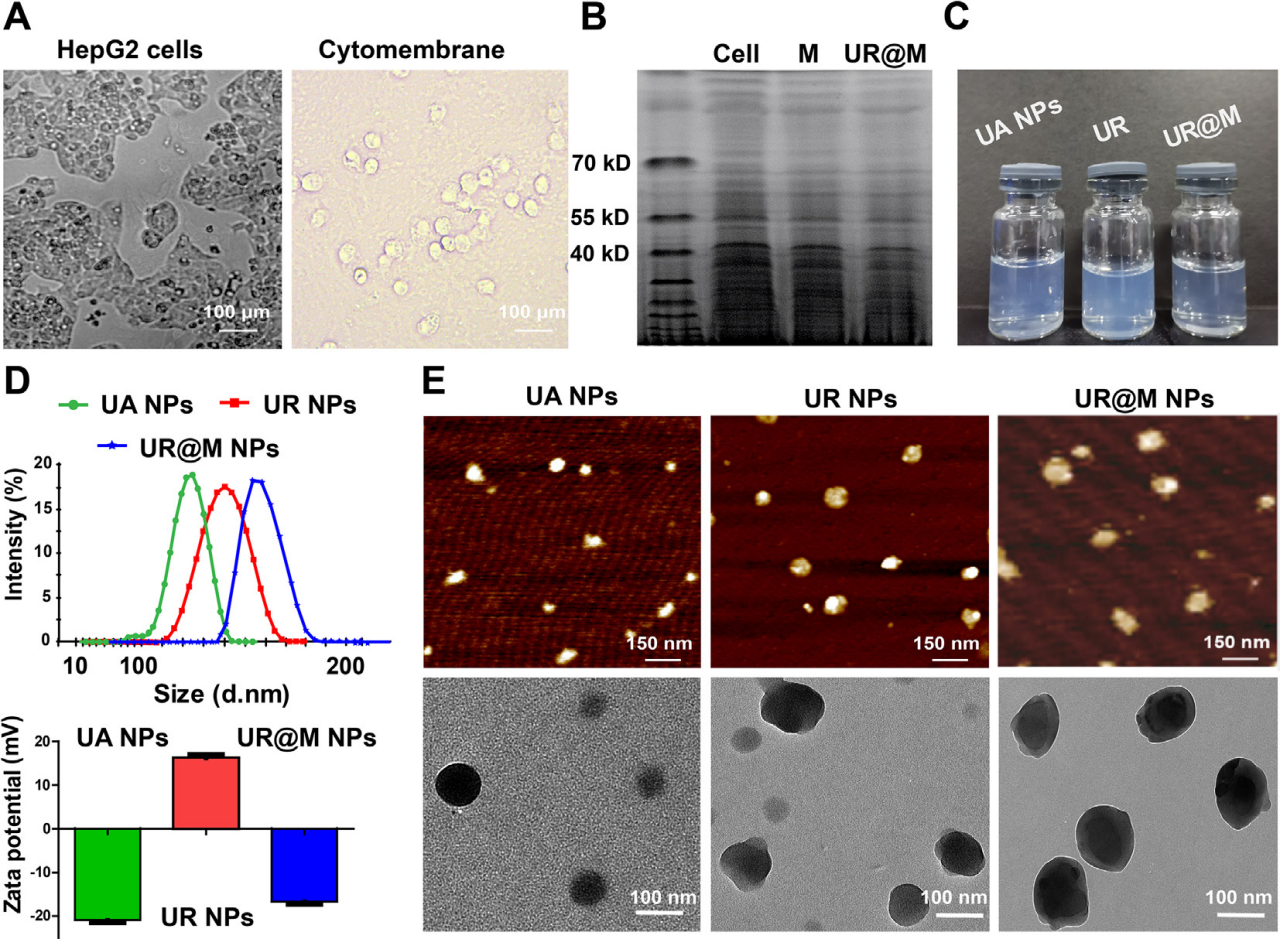
The preparation of UR@M NPs. (A) The images of HepG2 cells and the cell membrane. Scale bar = 100 μm. (B) SDS-PAGE protein analysis of biomimetic UR@M NPs. (C) The images of the UA NPs, UR NPs, and UR@M NPs solutions. (D) The particle size and zeta potential of nanodrugs. (E) AFM images and TEM images of the nanodrugs. AFM scale bar = 150 nm, TEM scale bar = 100 nm.

sgRNA with efficient biological activity was needed to ensure the efficient inhibition of PD-L1 expression and high gene-editing efficiency. Firstly, we designed the two *PD-L1* targeted sgRNA to construct the recombinant CRISPR/Cas9 plasmid of pX458 (Supporting Information Fig. S1). The gene expression of *PD-L1* was evaluated after the plasmid was transfected into HepG2 cells, and the result proved that both of the plasmids possessed biological activity with the evaluation of qRT-PCR and Western blots. By comparison, the sgRNA-2 exhibited better gene editing efficiency in inhibiting the expression of PD-L1 (Supporting Information Fig. S2). Therefore, sgRNA-2 was used to establish a one-step Cas9/RNP complex by binding with Cas9 protein as described in the Methods. Afterward, UA NPs were prepared as previously reported^21^, and the UA-template self-assembly system was used to combine the Cas9/RNP. The optimization of the self-assembly NPs is shown in Supporting Information Table S2. A mass ratio of 60:3:1 was deemed to be the best choice to prepare UR NPs, which exhibited a suitable particle size of 156.2 ± 2.6 nm with good dispersion. UR had a higher UA (78.40%) and Cas9/RNP (68.46%) encapsulation efficiency compared to the other optimum proposal. However, the particle size and stability of the self-assembly NPs were suboptimal without the introduction of LMWP (Supporting Information Fig. S3). Homologous HCC cancer cell membranes were obtained from HepG2 cells and had a transparent, round appearance (Fig. 1A). It was supposed that an increase in particle size and the charge reversal of UR NPs would be found after cell membrane coating, so an incubation under ultrasonication for 15 min was chosen to prepare UR@M NPs (Supporting Information Table S3). UR@M NPs exhibited a great advantage in UA (80.48%) and Cas9/RNP (4.26%) drug loading, almost constituting the entire nanodrug due to the self-assembly process. Sodium dodecyl sulfate-polyacrylamide gel electrophoresis (SDS-PAGE) suggested that the free cytomembrane and UR@M NPs were highly consistent in protein bands by Coomassie blue staining (Fig. 1B). The image revealed that almost all membrane proteins were retained on the UR@M NPs in the preparation.

UA NP, UR NP, and UR@M NP solutions were light blue with no impurities (Fig. 1C). The particle size was increased from UA NPs to UR@M NPs. In addition, surface zeta potential analysis showed that UR had a zeta potential of 16.8 ± 0.1 mV after Cas9/RNP and LMWP were added compared to the UA NPs (−20.4 ± 0.06 mV), whereas the zeta potential changed with cell membrane camouflage of UR@M (−17.2 ± 0.1 mV) (Fig. 1D). The negative charge of UR@M could reduce non-specific phagocytosis in the systemic circulation, while the positive charge of UR in the tumor environment induced better cellular uptake. The charge reversal of the nanodrug achieved more efficient targeted delivery *in vivo*. We further analyzed the morphology of the nanodrugs using an atomic force microscope (AFM), transmission electron microscope (TEM), and scanning electron microscope (SEM). All showed uniform sphere morphology (Fig. 1E and Supporting Information Fig. S4). UR@M displayed an obvious shell that formed around the nanodrugs compared to UR, confirming the successful fabrication of UR@M based on self-assembly and the biomimetic system.

### 3.2 Molecular interaction analysis and properties detection of nanodrug

**Figure 2:**
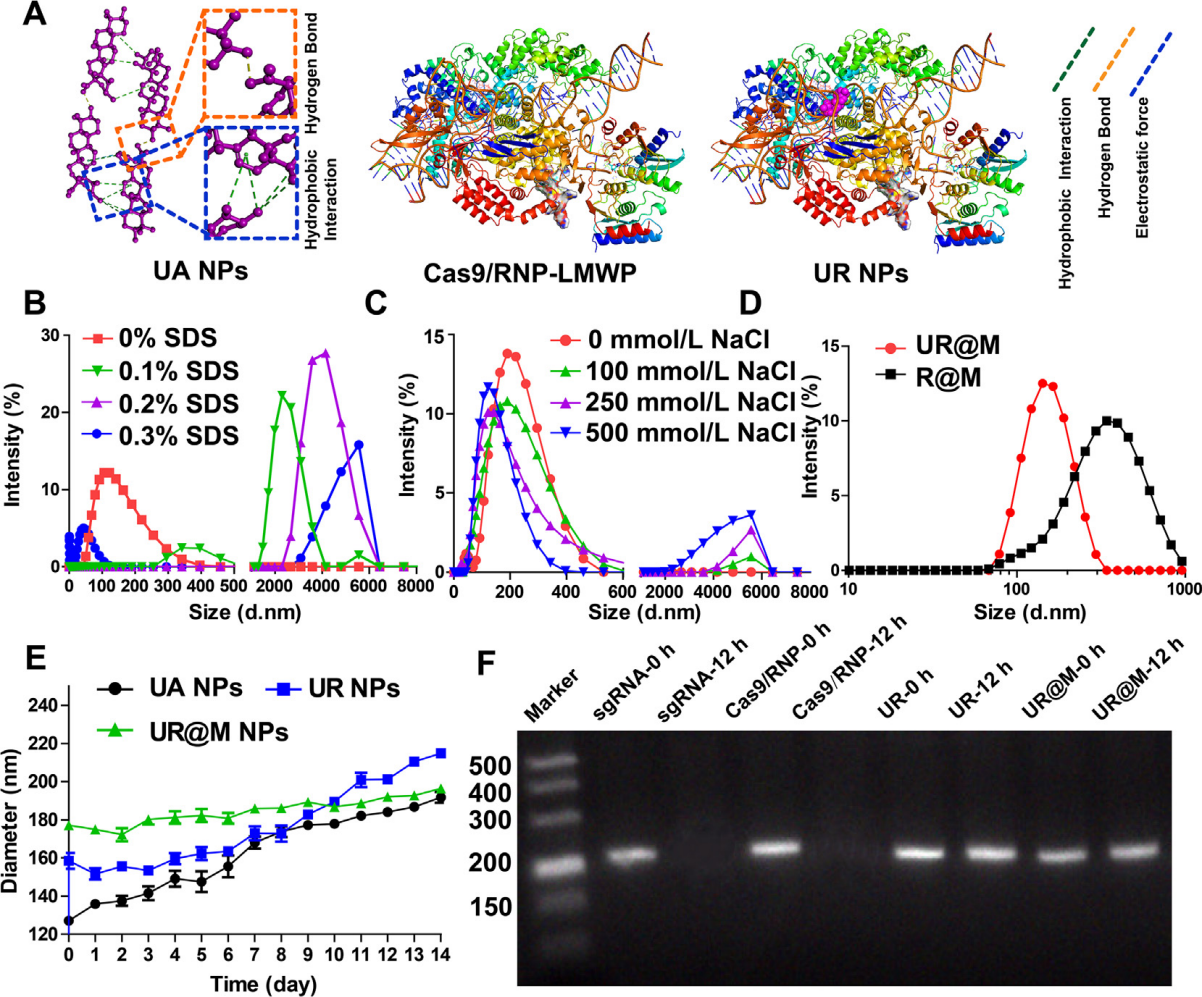
The molecular docking analysis and the stability of the UR@M NPs. (A) Molecular interaction analysis of UA NPs, Cas9/RNP-LMWP, and UR NPs. (B) The change of particle size of UR NPs when diluted by different concentrations of SDS and (C) NaCl. (D) The particle size of R@M compared to the UR@M NPs. (E) The monitoring of particle size to evaluate the stability of nanodrugs during in 7 days. Data are presented as mean ± SD (*n*=3). (F) The stability of sgRNA in UR@M NPs.

AutoDock was used to predict the possible interactions between UA, Cas9/RNP, and LMWP to explore the mechanism of the UA-templated self-assembly system. As shown in Fig. 2A, hydrophobic interactions and hydrogen bond interactions were mainly present in the UA-templated self-assembly, and electrostatic forces were responsible for the connection between Cas9/RNP and LMWP. Subsequently, several concentrations of SDS and NaCl were further added to the nanodrug to verify the hypothesis. As shown in Fig. 2B, SDS broke the hydrophobic interactions and hydrogen bond, the particle size of UR increased due to the self-assembled structure, and the level of depolymerization was SDS concentration dependent. The addition of NaCl had a similar result, except that there was still a part of the nanoparticles existing (Fig. 2C). This was caused by the change in charge, which affected the self-assembly of Cas9/RNP and LMWP, and the UA NPs remained intact nanostructures. Moreover, the Cas9/RNP and LMWP complex could not form a nanostructure by the coating of cytomembrane compared to the UR@M, the result demonstrated the necessity of UA in the self-assembly nanodrug (Fig. 2D).

The particle size of UA NPs, UR NPs, and UR@M NPs was recorded in 7 days to verify the stability of the nanodrug. The average UR and UR@M diameter was not significantly changed, while UA NPs exhibited a larger change (Fig. 2E). We used different solvents (PBS, NaCl, Dulbecco's modified Eagle medium (DMEM) + 10% fetal bovine serum, and serum) to investigate the stability of the nanodrugs in the physiological environments (Supporting Information Fig. S5). The result showed the good stability of UR and UR@M compared with UA NPs, as the previous conclusion. We also evaluated the stability of sgRNA in the nanosystem. As shown in Fig. 2F, free sgRNA and sgRNA in Cas9/RNP were degraded in the natural environment after 12 h. In comparison, the sgRNA in UR or UR@M showed good stability, and the UR@M NPs had better protection for sgRNA in avoiding the natural degradation of RNA in normal environment. We investigated the drug release of UA and Cas9 protein from UR NPs in serum. The results indicated the slow release of UR in the physiological environment, but the release rate was weak (Supporting Information Fig. S6). Subsequently, PBS at different pH values was tested. The nanodrugs were rapidly released in a pH 5.0 environment, and there was depolymerization and precipitation in the acidic environment (Supporting Information Fig. S7). The results suggested the good stability of the nanodrugs in a normal physiological environment, as well as efficient drug release by pH-responsive UR in an acidic tumor environment.

### 3.3 Cellular uptake of UR@M

**Figure 3:**
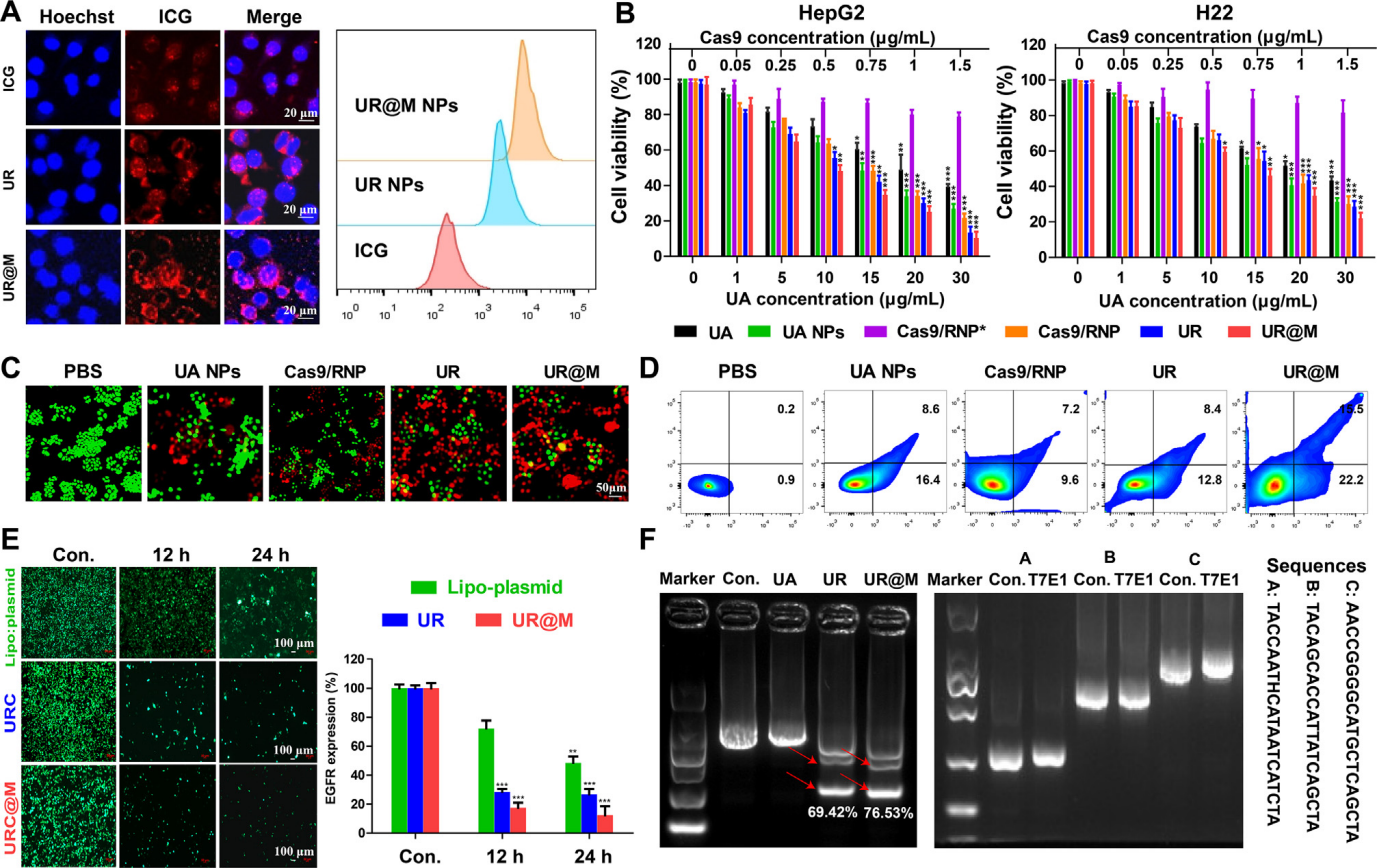
The detection of cellular uptake, cytotoxicity, and the gene editing efficiency of UR@M NPs. (A) The fluorescent images and flow quantification of HepG2 cells treated with nanodrugs. (B) Survival of HepG2 cells and H22 cells treated with different formulations (UA, UA NPs, Cas9/RNP∗, Cas9/RNP, UR NPs, UR@M NPs). The concentration of 0 μg/mL was treated as the control group for significance analysis. (C) The staining of living/dead cells after treated with different formulations. Scale bar = 50 μm. (D) The cell apoptosis of HepG2 cells was evaluated by flow cytometry. (E) The detection of green fluorescence in EGFP-expressing HEK-293T cells after the addition of nanodrugs with Lipo-transfection as the control. The fluorescence intensity of 0 h was regarded as control group for significance analysis. Scale bar = 100 μm. (F) The efficient gene therapy of PD-L1 and off-targeted effect of UR@M were examined by T7E1 and agarose gel electrophoresis. Data are presented as mean ± SD (*n*3). ∗*P* < 0.05, ∗∗*P* < 0.01, ∗∗∗*P* < 0.001.

ICG was used as a contrast agent to track the distribution of UR@M in tumor cells to investigate the enhancing effect of the biomimetic coating and LMWP for the cellular uptake of nanodrugs. Firstly, with free ICG as the control, the same fluorescence absorption peak was detected in UR and UR@M after dialysis, which proved the efficient self-assembly of amphiphilic ICG in the nanodrugs (Supporting Information Fig. S8). Then, the cellular uptake of UR@M NPs with UA NPs and UR NPs as the controls at the same drug concentration was evaluated by fluorescence imaging. As shown in Supporting Information Fig. S9, all of the groups showed a tendency to increase fluorescence intensity in HepG2 cells from 2 to 6 h, and then the fluorescence intensity gradually decreased. In comparison, UR NPs exhibited a rapid cellular uptake at 2 h compared to UA NPs, which confirmed the efficient cell membrane penetration of LMWP in the nanosystem. UR@M not only showed faster cellular uptake, but higher fluorescence intensity was observed by flow cytometry. HepG2 cells incubated with UR@M had a higher fluorescence intensity compared to UA NPs and UR NPs (Fig. 3A). The same result was also demonstrated by quantifying UA, where the concentration of UA in the UR@M group was found to be higher than in the other group (Supporting Information Fig. S10). The fluorescence of UA NPs was mainly seen in the cytoplasm of cells, while UR and UR@M moved into the nucleus at 6 h. The results confirmed the nuclear localization signal (NLS) in Cas9/RNP available for the application of CRISPR/Cas9 in gene therapy. Thereafter, H22 mouse liver cancer cells were used for the same experiment, and UR@M also showed a more significant fluorescence intensity, whether detected by CLSM or flow cytometry (Supporting Information Fig. S11). These results proved the quick and efficient cellular uptake of UR@M NPs and that the nanodrug entered the nucleus to ensure gene therapy using CRISPR/Cas9.

### 3.4 Cytotoxicity assay

First, the tumor cells were investigated for the cytotoxicity of drugs by the co-culture of Jurkat cells. Besides, free UA, free Cas9/RNP, UA NPs, and UR NPs, Cas9/RNP∗ containing a scrambled sequence instead of sgRNA-*PD-L1* was incubated with the cells as the control. As shown in Fig. 3B, Cas9/RNP∗ exhibited weak cytotoxicity with no special gene targeting. All of the other groups presented a dose-dependent inhibition of HepG2 cell and H22 cell proliferation. The self-assembled UA NPs showed higher cytotoxicity compared to free UA, and UR NPs were more cytotoxic than UA NPs and Cas9/RNP. The efficient gene editing of PD-L1 by Cas9/RNP enhanced the cytotoxicity of Jurkat cells to tumor cells. The degree of synergy between UA and Cas9/RNP in the self-assembled UR nanodrug was investigated by the combination index (CI). The results indicated the synergy of UA and PD-L1 gene therapy by CI values lower than 1 (Supporting Information Fig. S12). UR@M exhibited the highest cytotoxicity both in HepG2 cells and H22 cells compared to the control groups at the same drug concentration, with similar findings in Huh-7 cells (Supporting Information Fig. S13). In addition, the cytotoxicity of the same groups was also examined in normal live L02 cells, renal embryonic HEK-293T cells, and endothelial HUVEC cells. The results showed that the cytotoxicity of the nanodrugs in normal cells was much weaker than in tumor cells (Supporting Information Fig. S14). The findings provided evidence for the biosafety of UR@M.

Subsequently, live and dead cell staining and a cell apoptosis experiment were further performed to test the cytotoxicity of UR@M. As shown in Fig. 3C, the number of red dead HepG2 cells was significantly increased after incubation with the drugs, and the UR@M showed the strongest cytotoxicity compared to UA NPs, Cas9/RNP, and UR. The same result is shown in Fig. 3D. UR@M induced the most efficient cell apoptosis. In summary, UR@M exhibited synergistic therapy by UA and PD-L1 gene therapy on tumor cells, with low cytotoxicity to normal cells.

### 3.5 Gene editing efficiency of nanodrug

EGFP-expressing HEK-293T cells were used to quantify knock-out efficiency according to fluorescence to investigate the effectiveness of UR@M gene therapy, and the sgRNA of the *EGFP* gene was designed for the nanodrugs. As shown in Fig. 3E, the fluorescence intensity of cells incubated with UR and UR@M was observed by fluorescence microscopy, with Lipofectamine-transfection as the control. As the *EGFP* gene was knocked out by the CRISPR/Cas9 system, green fluorescence in the nanodrug groups was significantly decreased compared to the Lipo-transfection control at 12 and 24 h, indicating that UR@M showed better gene-editing efficiency than UR. Subsequently, efficient gene editing for *PD-L1* was examined in HepG2 cells. By the cut of the T7E1 enzyme, the knock-out of the gene could be analyzed according to the newly produced DNA bonds. As shown in Fig. 3F, both UR and UR@M exhibited a high *PD-L1* gene-editing efficiency of 69.42% and 76.53%, respectively. Potential off-target genes were also detected to evaluate the biosafety of UR@M. It confirmed that there was no wrong knock-out occurring in the gene editing. Therefore, UR@M could be used for *in vivo* research based on the potent gene therapy effects of *PD-L1*.

### 3.6 The activation of innate immune system of UR@M *in vitro*

**Figure 4:**
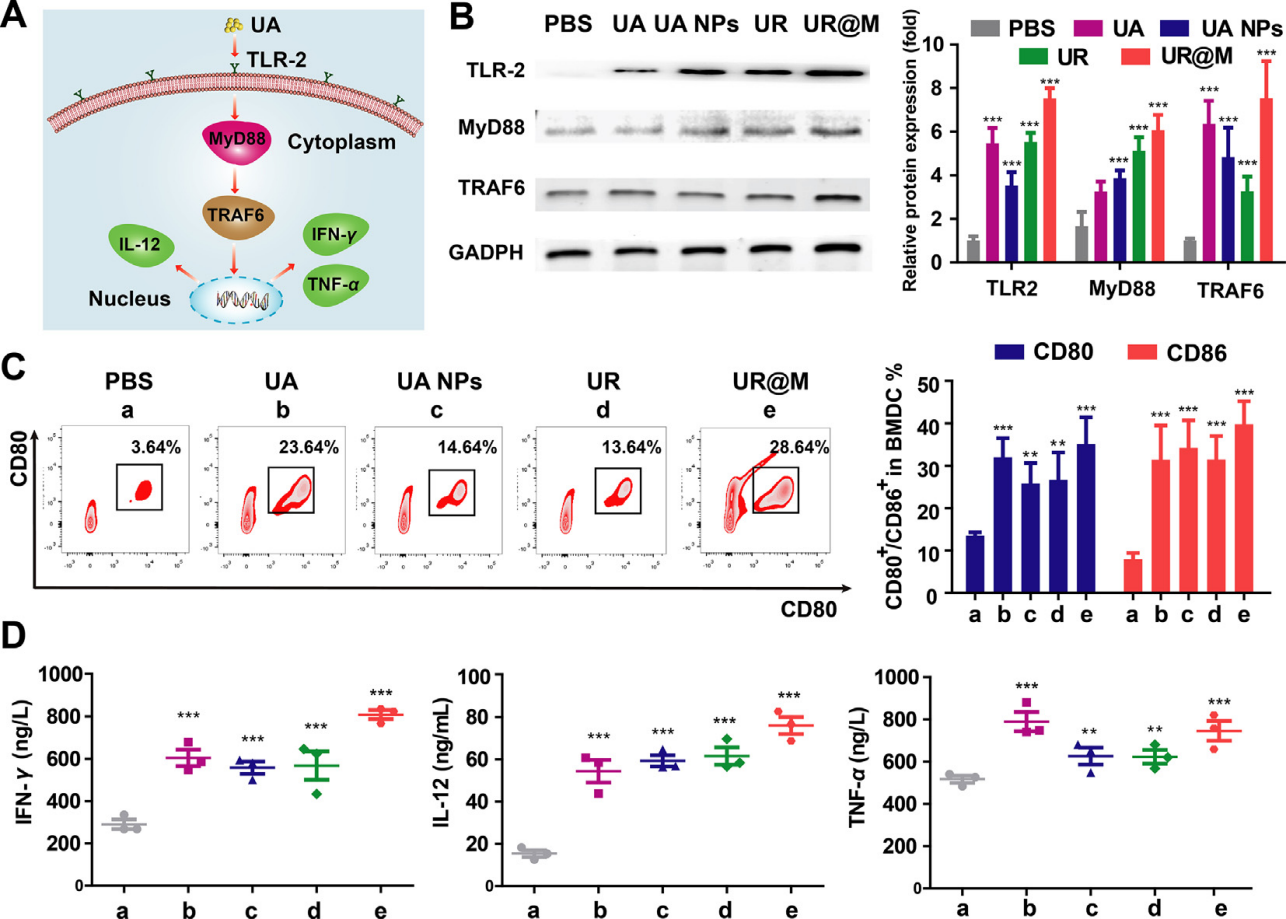
The activation of innate immune system by UR@M *in vitro.* (A) Schematic illustration of the activation of TLR-2 pathway in immune cells. (B) The protein and mRNA expression of TLR-2-MyD88-TRAF6 in PBMCs treated with UA, UA NPs, UR NPs, and UR@M NPs. (C) The quantification of maturation DC cells in BMDC by flow cytometric plots and related marker expression (CD80^+^, CD86^+^). (D) Representative cytokine expression of IFN-*γ*, IL-12 and THF-*α* were examined by ELISA. PBS group was treated as the control for significance analysis. Data are presented as mean ± SD (*n*=3). ∗*P* < 0.05, ∗∗*P* < 0.01, ∗∗∗*P* < 0.001.

The PBMCs of tumor-bearing mice were collected to evaluate the potential target and molecular mechanism of UA. As shown in Supporting Information Fig. S15, several receptors of the innate immune pathway were investigated after the PBMCs were treated with the free drug and nanodrugs. With the activator of the relevant pathway as the positive control, UA exhibited significant activation for TLR-2. A similar induction effect of UA was also found in the UR NPs and UR@M NPs. Therefore, the TLR-2-MyD88-TRAF6 pathway and related innate immune system could be activated by UR@M NPs (Fig. 4A). Afterward, we further confirmed the efficient activation of UR@M NPs to the pathway by the expression of protein (Fig. 4B). Additionally, we investigated that bone marrow-derived dendritic cells (BMDCs) incubated with UR@M NPs displayed a substantial increase in expression of the CD80 and CD86, suggesting that UA in UR@M NPs played the main role in the activation of innate immune system (Fig. 4C). The pathway induced the production of multiple cytokines associated with the immune system, and the high expression of IFN-*γ*, IL-2, and TNF-*α* was detected with the addition of UA, UA NPs, UR NPs, and UR@M NPs (Fig. 4D). Overall, these results demonstrated that UR@M NPs could significantly activate TLR-2 pathway and promote the maturation of DC cells, and the UR@M showed the strongest increase of the cytokines.

### 3.7 *In vivo* experiment

**Figure 5:**
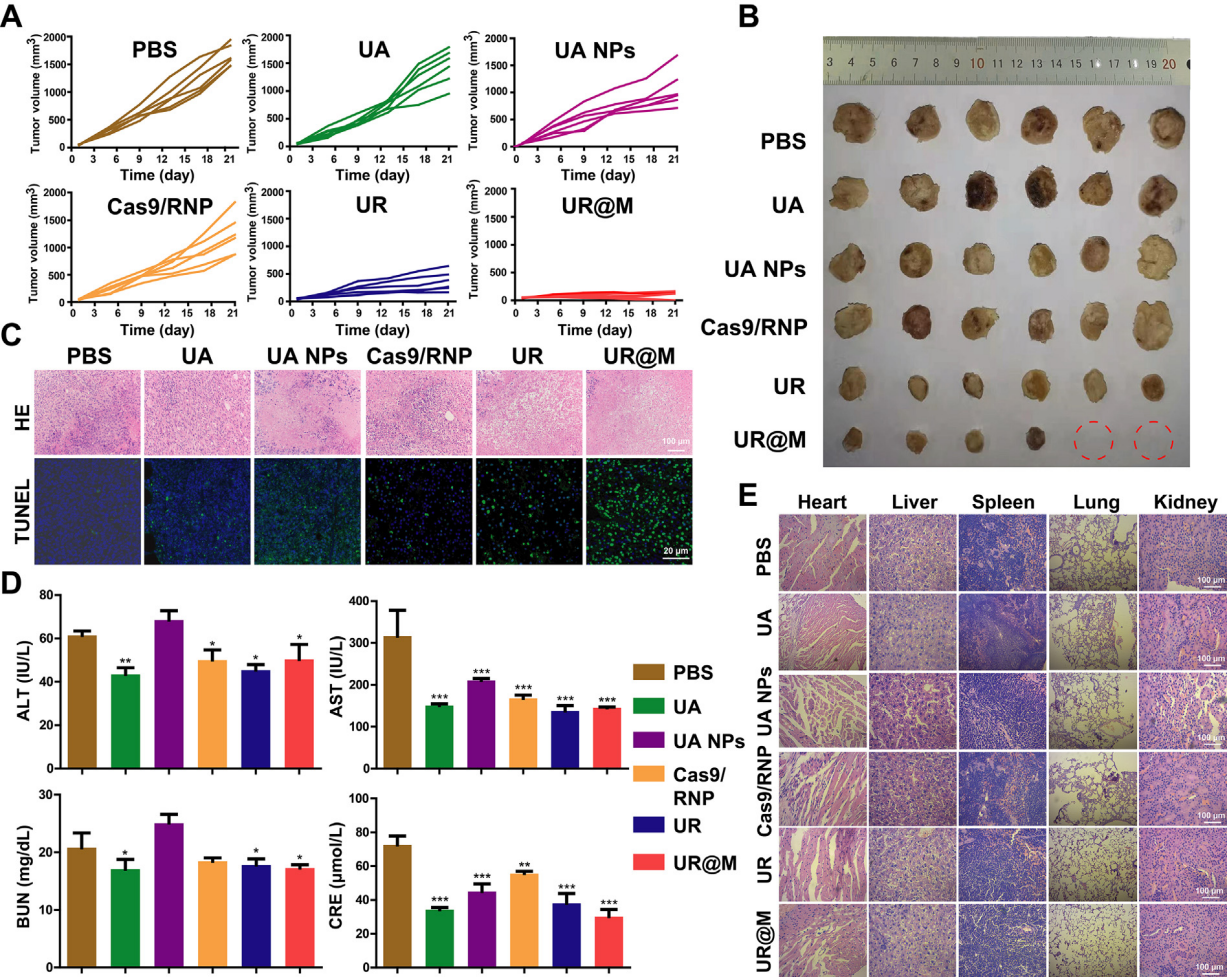
The evaluation of the anti-tumor effect of UR@M NPs *in vivo*. (A) HCC tumor growth curves of different groups under the treatments (*n* = 6). (B) The image of excised tumors after the treatment. (C) H&E staining and TUNEL assay of tumor tissues. H&E scale bar = 100 μm, TUNEL scale bar = 20 μm. (D) Blood biochemistry of mice after the treatment. PBS group was treated as the control for significance analysis. (E) H&E staining of major organs of tumor-bearing mice following the indicated treatments. Scale bar = 100 μm. Data are presented as mean ± SD (*n*=3). ∗*P* < 0.05, ∗∗*P* < 0.01, ∗∗∗*P* < 0.001.

Subcutaneous transplantation and orthotopic implantation tumor-bearing animal models were applied to evaluate the *in vivo* treatment of UR@M. The therapy of different formulations was performed once in two days, and the volume of subcutaneous tumors was recorded during the treatment by intravenous injection in 21 days as shown in Fig. 5A. Particularly, the tumors of two mice maintained in a small state during the treatment by injecting UR@M, and the two tumors almost disappeared at about 18 days (Fig. 5B). With the PBS, UA, UA NPs, and Cas9/RNP as the control, the nanodrugs of UR and UR@M exhibited more efficient inhibition in tumor growth, and the UR@M NPs had the best tumor inhibitory rate after the treatment (Supporting Information Fig. S16). The gene therapy of CRISPR/Cas9 based on the self-assembly system was also investigated in tumors, the significant knock-out of PD-L1 was found in UR- and UR@M-treated groups, and the UR@M group showed a great gene-editing efficiency of 64.42% (Supporting Information Fig. S17). Meanwhile, H&E staining showed obvious tumor tissue damage in the UR@M therapy group compared to the control groups, and same result was proved in the tumor tissue by the TUNEL assay (Fig. 5C). In addition, the slow gain of body weight was found in PBS-treated tumor-bearing group compared to the healthy mice of wild type (Supporting Information Fig. S18). In comparison, mice of the UR@M therapy group exhibited a normal body weight change as the wild type. Subsequently, by counting the number of tumor nodules in the liver, the therapeutic effect of nanodrugs could be evaluated. As shown in Supporting Information Fig. S19, the UR@M showed the best inhibition of tumor growth in liver compared to the free drugs and UR. The PBS group showed significant weight loss, while the UR@M group maintained normal weight gain. It indicated that orthotopic tumor caused obvious damage to the body, and UR@M significantly reduced the harm of tumor to the liver and had good safety. We further explored the safety of the UR@M in the following experiment.

The values of alanine aminotransferase (ALT), aspartate transaminase (AST), creatinine (Cre) and blood urea nitrogen (BUN) were examined to evaluate the damage of liver and kidney. As shown in Fig. 5D, the biochemical blood markers for organ injury were decreased in all of the therapy groups, especially for the UR@M. H&E staining was performed to evaluate the histological damage of heart, liver, spleen, lung, and kidney. The results confirmed that UR@M had no obvious toxicity to several organs (Fig. 5E). In addition, the hemolysis assay of UR@M NPs was performed by mixing the red blood cells with the same drug concentration *in vivo*. With the water and PBS regarded as the positive and negative control, the hemolysis ratio of UR@M was less than 5%, which showed the nanoparticles had no hemolysis effect (Supporting Information Fig. S20). These results demonstrated the effective therapeutic effect of UR@M NPs *in vivo* with satisfied biocompatibility and biosafety.

### 3.8 *In vivo* fluorescence imaging

**Figure 6:**
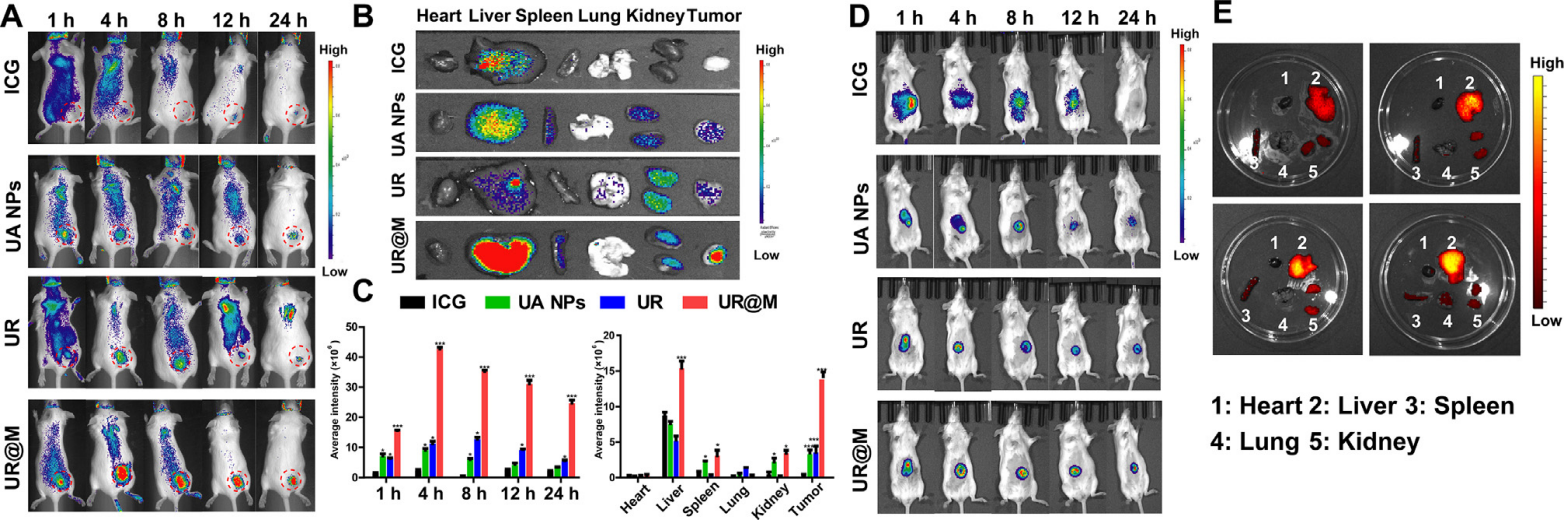
*In vivo* fluorescence images of mice in free ICG and NPs-treated groups. (A) The fluorescence images of free ICG and nanodrugs injection groups recorded in 24 h. (B) The fluorescence images of major organs and tumors after the sacrifice of mice. (C) Fluorescence intensity of tumor in living imaging and the excised major organs. (D) Fluorescence images of live mice and (E) excised tissues in orthotopic tumor models. The free ICG was treated as control group for significance analysis. Data are presented as mean ± SD (*n*=3). ∗*P* < 0.05, ∗∗*P* < 0.01, ∗∗∗*P* < 0.001.

The fluorescence imaging of nanodrugs in the HCC animal model was evaluated in 24 h. Compared to the fluorescence imaging of control group ICG was distributed in body without the specificity, the UA NPs, UR NPs, and UR@M NPs showed enhanced drug accumulation in tumors. Meanwhile, UR@M exhibited the best tumor-targeting and long-circulation effect *in vivo.* The fast drug accumulation of UR@M was found at 1 h, and the significant tumor location fluorescence was still observed at 24 h (Fig. 6A). In comparison, the tumor accumulation of UA NPs and UR NPs were mainly caused by the EPR effect, and the UR@M showed better tumor active targeting owing to the coating of HCC homologous cytomembrane. The mice were sacrificed after the fluorescence imaging *in vivo*, the tumors and the major organs were collected. The distribution of fluorescence in the excised tissues confirmed that the nanodrug was metabolized mainly by the liver and kidneys, and UR@M significantly enhanced the drug accumulation (Fig. 6B). Moreover, the average fluorescence intensity of tumor also showed more efficient tumor accumulation of UR@M compared to the free ICG and nanodrugs of UA and UR at different time points (Fig. 6C). The fluorescence tumor/liver rate of UR@M was higher than that of free ICG and UR groups (Supporting Information Fig. S21). It indicated the good drug tumor accumulation of UR@M *in vivo*.

In addition, the efficient drug accumulation of UR@M NPs orthotopic tumors was also investigated by *in vivo* fluorescence imaging. As shown in Fig. 6D, the obvious fluorescence of UA NPs, UR NPs and UR@M NPs at liver were observed compared to the control group of free ICG. By comparison, UR@M group exhibited the most effective drug accumulation, and significant fluorescence was still present in liver at 24 h. The same result was confirmed in the excised organs as shown in Fig. 6E, the fluorescence of liver was higher in UR@M-treated group. These experiments proved the great targeting specificity and long circulation of UR@M.

### 3.9 Evaluation of synergistic immunotherapy of UR@M

**Figure 7:**
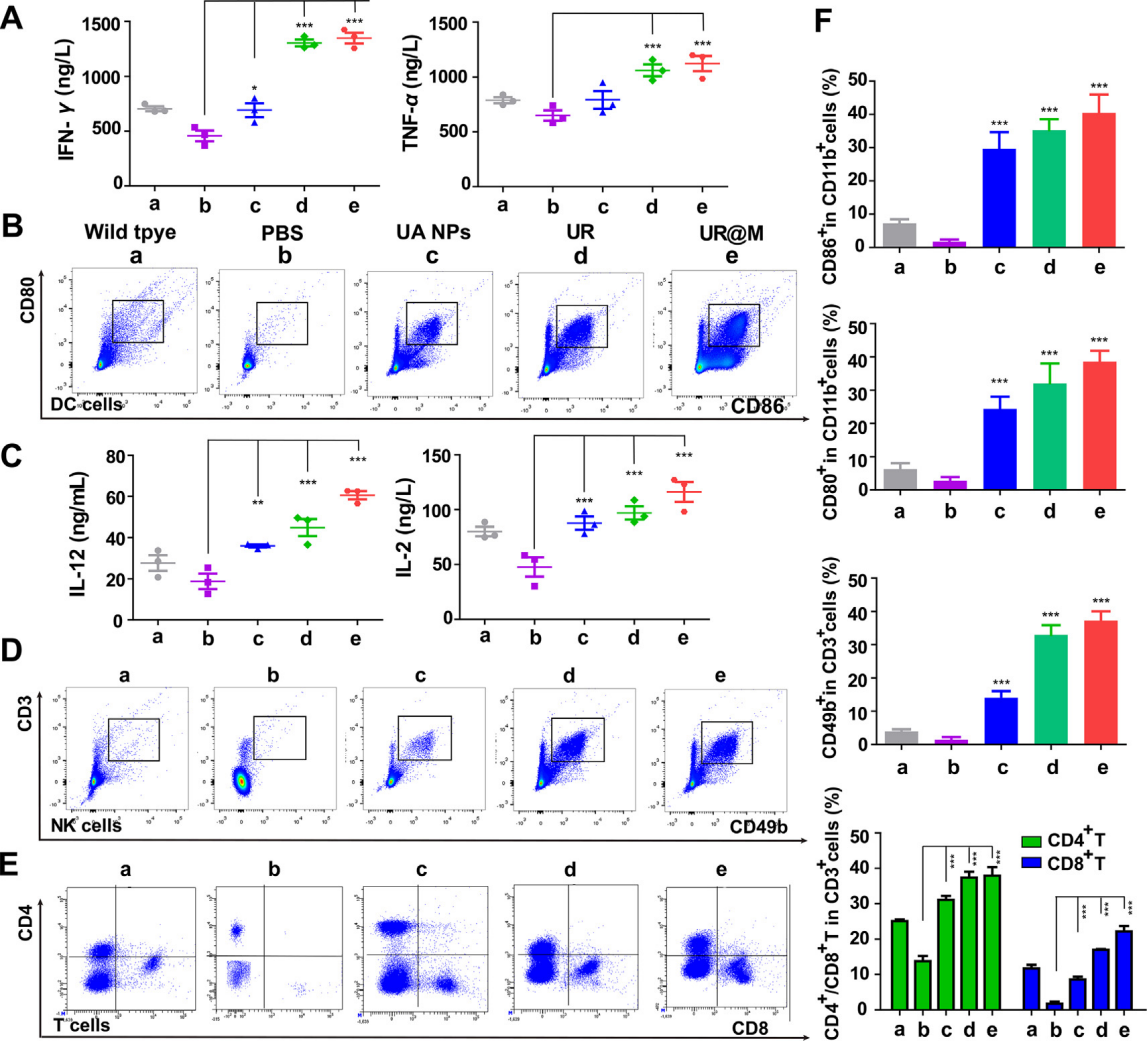
Assessment of immunotherapy of UR@M NPs for HCC treatment. (A) Serum cytokine concentrations of IFN-*γ* and TNF-*α* in different groups. (B) The maturation of DC cells was detected by flow cytometry. (C) Serum cytokine concentrations of IL-12 and IL-2 after the treatment. (D) The activation of NK cells, and CD4^+^/CD8^+^ T cells (E) induced by UR@M NPs. (F) The quantitation of related immune cells. PBS group was regarded as control for significance analysis. Data are presented as mean ± SD (*n*3). ∗*P* < 0.05, ∗∗*P* < 0.01, ∗∗∗*P* < 0.001.

As the efficient immune regulation of UR@M was proved by TLR-2 pathway and DC cells maturation, we further investigated the immunotherapy of the nanodrug *in vivo.* The increase of TNF-*α* and IFN-*γ* in serum after the treatment by UR@M NPs was associated with DC cells (Fig. 7A), and the co-expression of cell surface specific proteins (CD80, CD86) indicated the maturation of DC cells was significantly activated by the UA and nanodrugs (Fig. 7B). Subsequently, we also found that the IL-12 and IL-2 were induced by UR@M (Fig. 7C), the cytokines were involved in the production of NK cells. The result confirmed the correlation, and the UR@M exhibited the better effect than other groups in the activation of NK cells (Fig. 7D). It suggested that the UR@M NPs not only induced the activation of TLR-2 natural immune pathway *in vitro*, but showed significant regulation of innate immune *in vivo*, effectively inducing the generation of mature DC cells and NK cells. NK cells could directly participate in the killing of tumor cells, and DC cells participated in the presentation of specific tumor immune antigens, resulting in the enhancement of the immunotherapy of cytotoxicity T cells (CTLs).

Considering the effective inhibition of PD-L1 expression by UR@M in tumor tissue as proved by immunofluorescent staining (Supporting Information Fig. S22), the synergistic immunotherapy of UR@M could be a combination effect based on innate and acquired immunity for the inhibition of tumor growth. Notably, the percentage of CD4^+^ or CD8^+^ positive T cells from the UR and UR@M group remarkably increased compared with the tumor-bearing mice (Fig. 7E and F). By comparison, UR@M exhibited the most efficient enhancement compared to the other groups. In addition, the normal ratio of CD4^+^/CD8^+^ ranged from 1.4 to 2.0, the ratio was higher than the normal range in the tumor-bearing group with PBS treated, while for the UR@M NPs group, the numeric value was close to the group of wild type (Supporting Information Fig. S23). Finally, the percentage of CTLs in the tumor was investigated, the result provided visual evidence that the UR@M activated the immunity system and induced the tumor-infiltrating for HCC treatment (Supporting Information Fig. S24). These studies demonstrated the great synergistic immunotherapy of UR@M based on the UA and *PD-L1* gene editing through the combination of innate immunity and acquired immunity for HCC treatment.

## 4 Conclusions

In this work, we successfully constructed a co-delivery nanosystem based on UA-templated self-assembly for binding with the Cas9/RNP complex of CRISPR/Cas9 and cell transmembrane peptide. The universal template was used not only for the self-assembly of small molecule drugs but also for proteins. The nanodrug UR@M exhibited good stability and sgRNA protection, and the efficient cellular uptake and tumor accumulation were further enhanced by the homologous targeting of the cytomembrane coating. UR@M effectively inhibited HCC cell proliferation and apoptosis and induced the high gene-editing efficiency of *PD-L1*. The synergistic immunotherapy of UR@M was explored by activating the innate immune pathway of TLR-2-MyD88-TRAF6 pathway and *PD-L1* gene knock-out. The maturation of DC cells promoted the presentation of tumor immunogenic antigens, and the NK cells and CTLs induced the efficient immunotherapy, resulting in an excellent therapeutic effect on subcutaneous and orthotopic tumor models. This strategy based on a self-assembly nanodrug and the CRISPR/Cas9 system could provide a potential option for HCC treatment.

## Author contributions

Bing-chen Zhang and Jing-wei Shao designed the research. Bing-chen Zhang and Chun-mei Lai carried out the experiments and performed data analysis. Bang-yue Luo participated in part of the experiments. Bing-chen Zhang and Chun-mei Lai wrote the manuscript. Jing-wei Shao revised the manuscript. All of the authors have read and approved the final manuscript.

## Conflicts of interest

The authors declare no conflicts of interest.

## Appendix A Supporting information

The following is the Supporting Information to this article:
