# Supplementary material for: Triterpenoids-templated self-assembly nanosystem for biomimetic delivery of CRISPR/Cas9 based on the synergy of TLR-2 and ICB to enhance HCC immunotherapy
Source: Acta Pharm Sin B. 2024 May 8;14(7):3205–17. doi: 10.1016/j.apsb.2024.04.033 (PMC11252477; doi:10.1016/j.apsb.2024.04.033)
Supplement: Multimedia component 1 [file mmc1.pdf]

Supporting Information for

ORIGINAL ARTICLE

Triterpenoids-templated self-assembly nanosystem for biomimetic delivery of CRISPR/Cas9 based on the synergy of TLR-2 and ICB to enhance HCC immunotherapy

Bingchen Zhang[a,b,†], Chunmei Lai[a,†], Bangyue Luo[a], Jingwei Shao[a,*]

[a]*Fujian Provincial Key Laboratory of Cancer Metastasis Chemoprevention and Chemotherapy, College of Chemistry, Fuzhou University, Fuzhou 350116, China*

[b]*Department of Laboratory Medicine, Dongguan Institute of Clinical Cancer Research, the Tenth Affiliated Hospital of Southern Medical University (Dongguan People's Hospital), Dongguan 523058, China*

Received 6 December 2023; reviewed in revised form 17 March 2024; accepted 20 March 2024

[*]Corresponding author.

E-mail address: shaojw12@163.com (Jing-wei Shao).

[†]These authors made equal contributions to this work.

Table S1. Oligonucleotide sequence

Oligo Name	Sequence (5’ to 3’)
SgRNA-1 PD-L1 (Human)	GTCCAGATGACTTCGGCCTT
SgRNA-2 PD-L1 (Human)	TACCGCTGCATGATCAGCTA
TLR-2-F	GTGACTGCTCGGAGTTCTC
TLR-2-R	CTTCCTGCCTTCACTTGGT
TLR-3-F	TCCCAGCCTTACAGAGAAGC
TLR-3-R	CCTGTGAGTTCTTGCCCAAT
TLR-4-F	TGGAAGTTGAACGAATGGAATGT
TLR-4-R	ACCAGAACTGCTACAACAGATAC
TLR-6-F	CATCCTATTGTGAGTTTCAGGCAT
TLR-6-R	GCTTCATAGCACTACATCCCAAG
cGAS-F	GTCGGAGTTCAAAGGTGTGGA
cGAS-R	GACTCAGCGGATTTCCTCGTG
RIG-1-F	GAGCACTGGTGGATGCCTTA
RIG-1-R	GCTGTCTCTGTTGGTTCGGA
SgRNA PD-L1 (Mouse)	GCTTGCGTTAGTGGTGTACT

Table S2. The optimization of UR NPs. Data are presented as mean±SD (n=3).

Mass Ratio (UA: Cas9/RNP: CPP)	Size/nm	Zeta/mV	PDI	UA Encapsulation efficiency (%)	Cas9/RNP Encapsulation efficiency (%)
60:1:1	283.2± 12.5	11.7± 0.2	0.5± 0.1	63.2± 2.6	46.8± 3.4
60:2:1	239.1± 3.4	8.7± 0.3	0.7± 0.1	74.5± 5.8	58.5± 1.6
60:3:1	156.2± 2.6	16.8± 0.1	0.2± 0	78.4± 3.6	63.4± 2.8
60:4:1	687.7± 14.2	16.6± 0.2	0.2± 0	50.6± 6.2	26.4± 4.8
60:5:1	320.1± 8.6	13.4± 0.2	0.3± 0	68.6± 4.2	38.8± 5.2

Table S3. The optimization of UR@M NPs. Data are presented as mean±SD (n=3).

Ultrasound Time (min)	Size	PDI	Zeta/mV	UA Durg loading (%)	Cas9/RNP Durg loading (%)
5	152.4± 2.6	0.4± 0.0	16.1± 0.3	73.4± 4.2	2.3± 0.3
10	158.6± 3.2	0.2± 0.0	14.6± 0.2	76.8± 2.4	1.7± 0.6
15	178.2± 2.2	0.1± 0.0	-17.2± 0.1	80.4± 2.8	4.3± 0.2
20	196.8± 5.5	0.6± 0.1	-20.5± 1.4	72.6± 3.7	3.8± 0.5

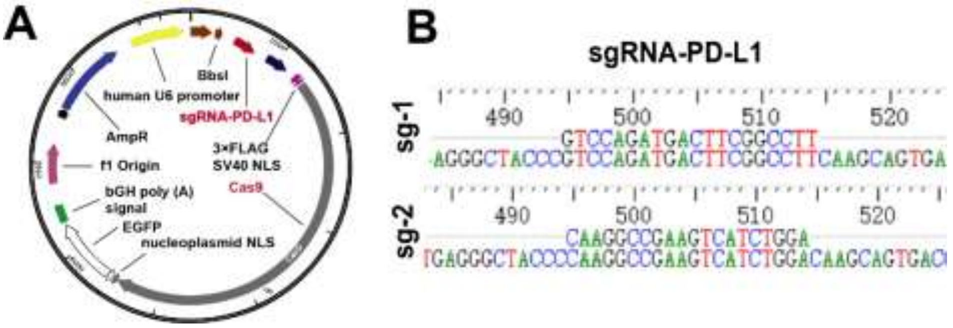

Figure S1. Construction of CRISPR/Cas9 recombinant plasmid. (A). The plasmid profile of recombinant PD-L1 targeted plasmid (pX458). (B). The verification of recombinant plasmid by sequence analysis.

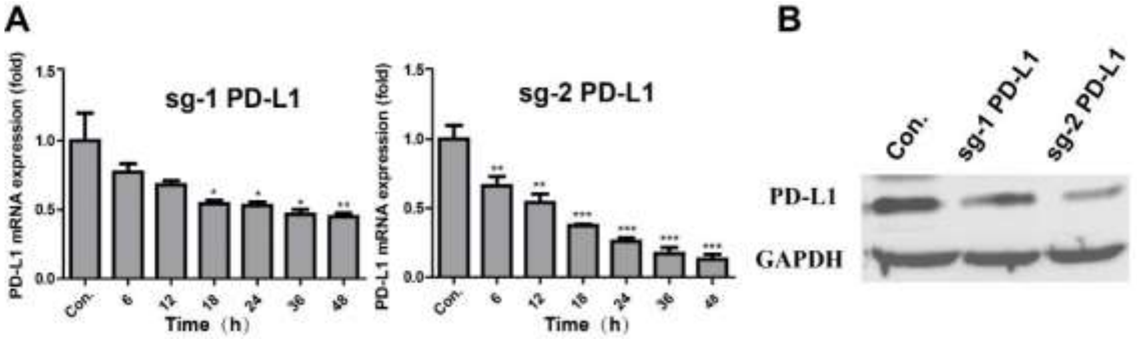

Figure S2. The detection of biological activity of designed sgRNA (A). The mRNA expression of PD-L1 in HepG2 cells after being treated with recombinant plasmid by lipo2000-transfection during 48 h. (B). The same recombinant plasmid transfection was performed after 48 h, and the protein expression of PD-L1 was examined by the Western blot. Data are presented as mean±SD (*n*=3). (*) $P < 0.05$, (**) $P < 0.01$, (***) $P < 0.001$.

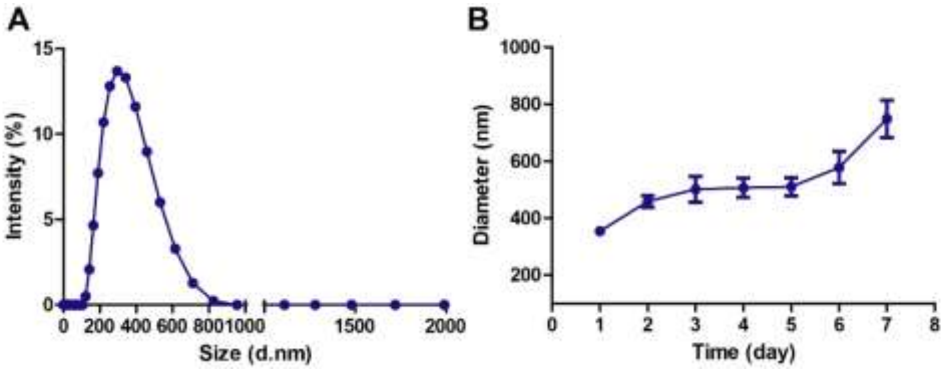

Figure S3. The particles size and stability of nanoparticles formed by the self-assembly of UA and Cas9/RNP without the introduction of LMWP. Data are presented as mean±SD (n=3).

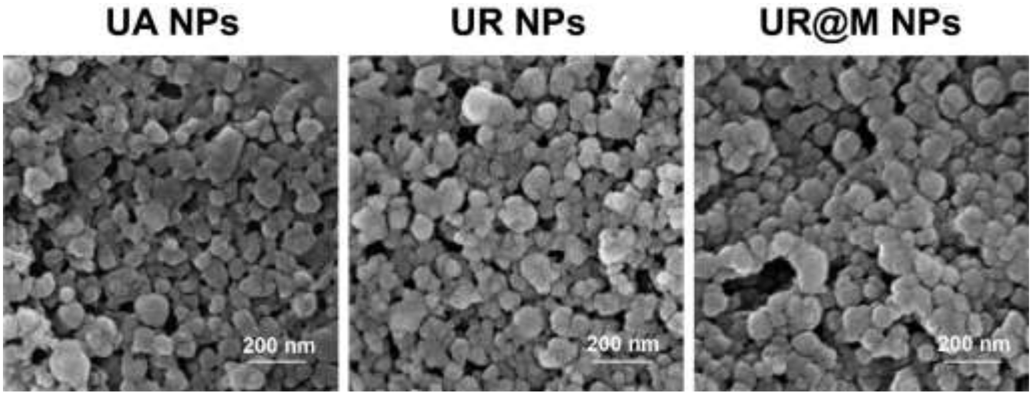

Figure S4. The scanning electron microscope (SEM) images of UA NPs, UR NPs, and UR@M NPs. Scale bar = 200 nm.

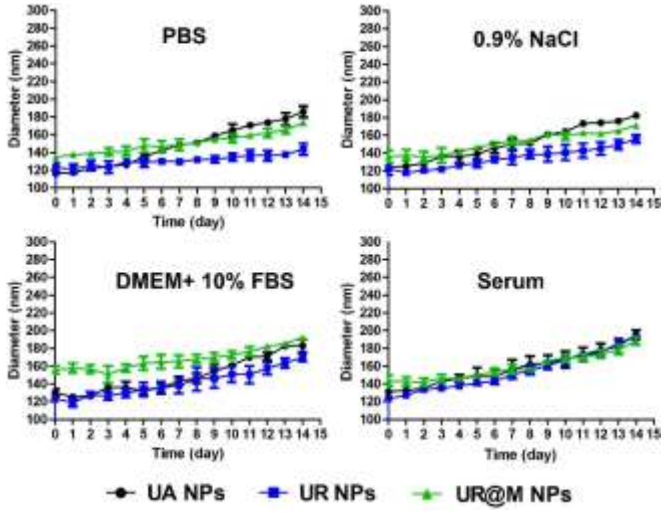

Figure S5. Evaluation of the stability of nanodrugs in a physiological environment. The size particles were detected 14 days after the UA NPs, UR NPs and UR@M NPs dissolved in different solutions compared to Day 0. Data are presented as mean±SD (n=3).

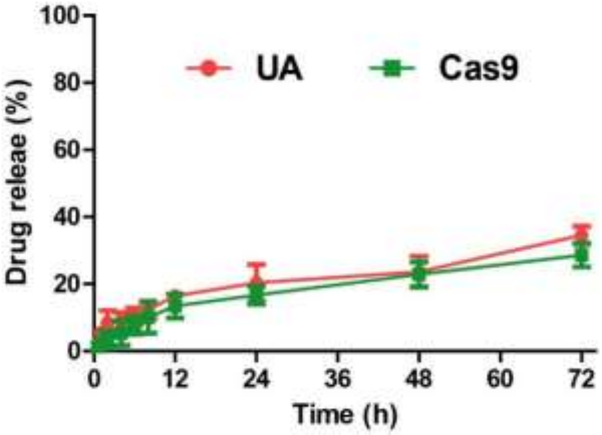

Figure S6. The UA and Cas9 release of UR in serum. Data are presented as mean±SD (n=3).

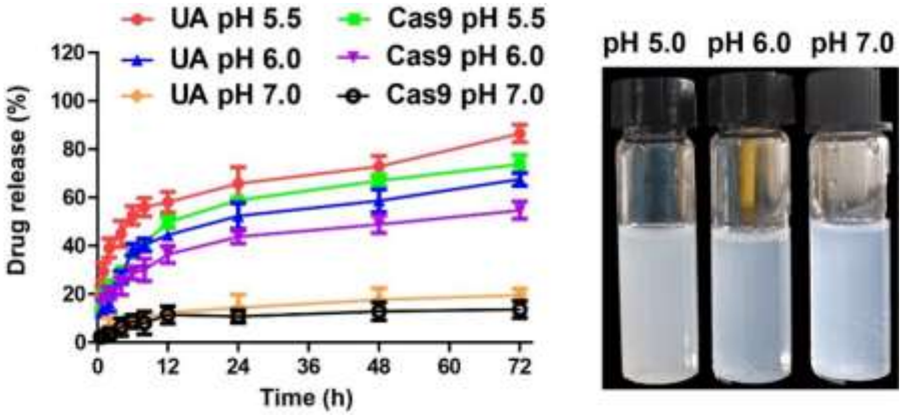

Figure S7. The drug (UA, Cas9) release of different pH values on UR NPs in 72 h. Data are presented as mean±SD (n=3).

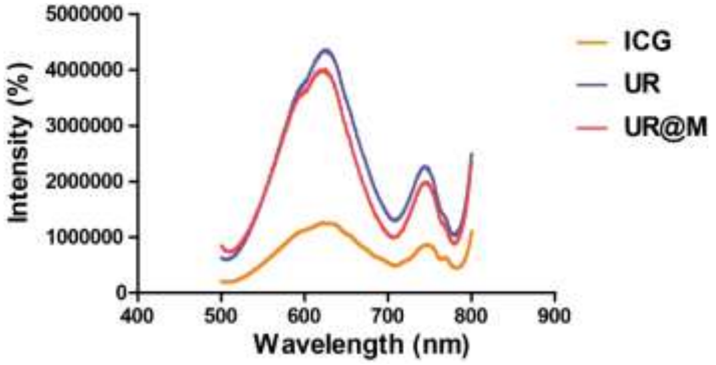

Figure S8. With the free ICG as the control, the fluorescence curve of UR and UR@M after the binding of ICG.

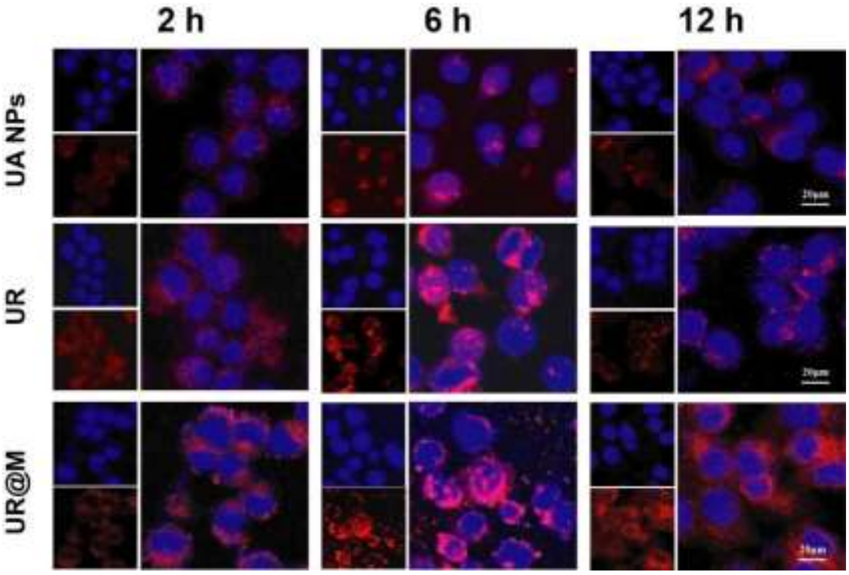

Figure S9. Fluorescent images of UA NPs, UR NPs and UR@M NPs in HepG2 cells at 2, 6, and 12 h. Scale bar= 20 μm.

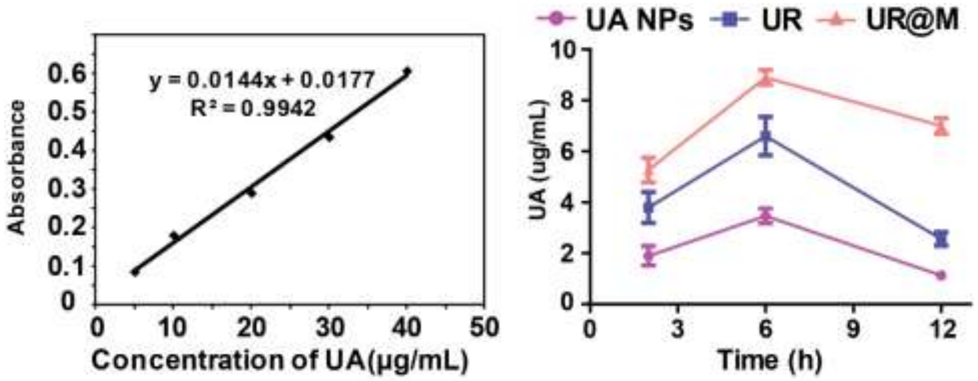

Figure S10. The quantification of UA in HepG2 cells at 2, 6, and 12 h after treated with UR@M NPs compared to the UA NPs and UR NPs. Data are presented as mean±SD (n=3).

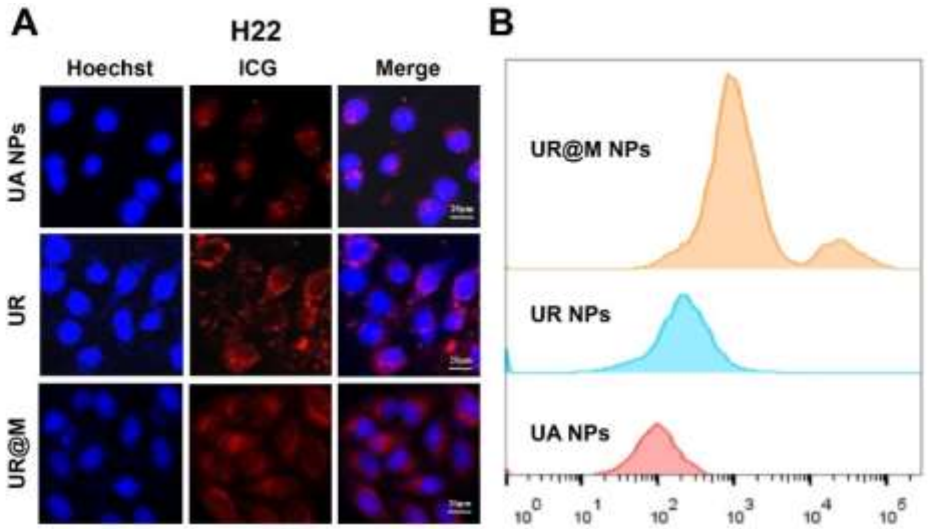

Figure S11. Cellular uptake of UR@M NPs in H22 cells. (A). Fluorescent images of cells after incubation with UA NPs, UR NPs, and UR@M NPs for 6 h. (B). The fluorescence intensity detection of cells by flow cytometry. Scale bar= 20 μm.

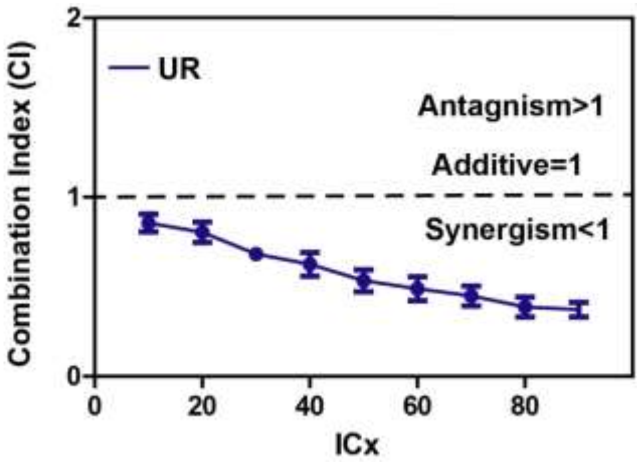

Figure S12. Combination index (CI) of CRISPR system and drug co-delivery nanosystem (UR NPs) compared to the single Cas9/RNP or UA when treated with HepG2 cells. CI values < 1 indicated synergism, CI values = 1 indicated an additive effect, and CI values > 1 indicated antagonism. Data are presented as mean±SD (n=3).

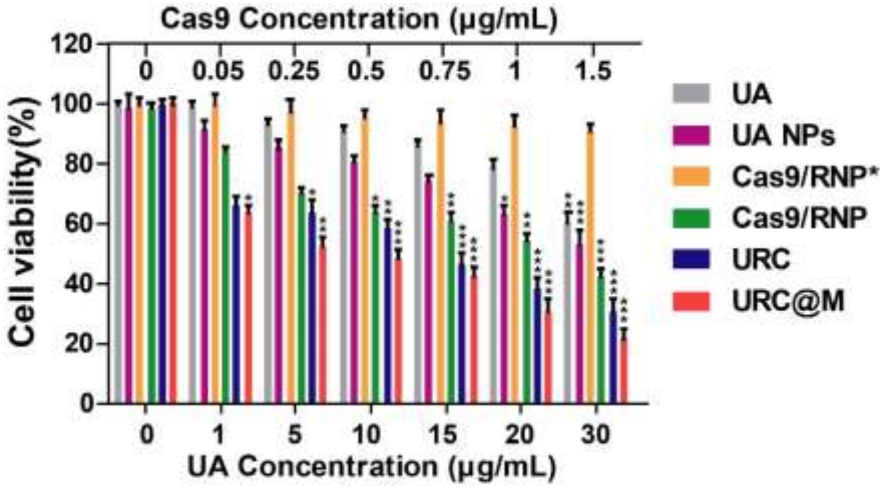

Figure S13. Survival of Huh-7 cells treated with different formulations (UA, UA NPs, Cas9/RNP*, Cas9/RNP, UR NPs, UR@M NPs). The concentration of 0 μg/mL was treated as the control group for significance analysis. Data are presented as mean±SD (*n*=3). $^{*}P < 0.05$, $^{**}P < 0.01$, $^{***}P < 0.001$.

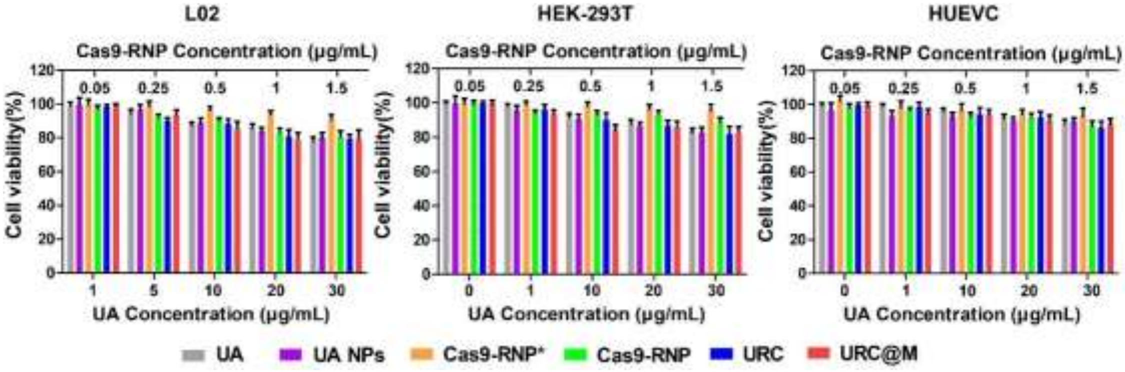

Figure S14. The normal cells of L02, HEK-293T and HUVEC were treated with different drugs with various concentrations. Cell viability was measured by CCK-8 assay. Data are presented as mean±SD (n=3).

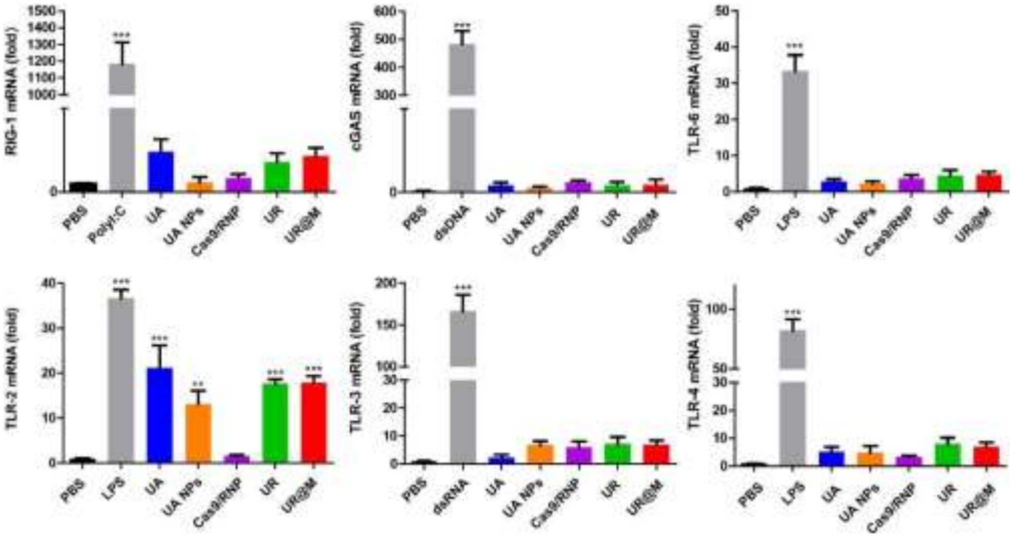

Figure S15. The mRNA expression of TLR2, TLR3, TLR4, TLR6, cGAS, and RIG-1 after being treated with different drugs in peripheral blood mononuclear cells (PBMCs). PBS group was treated as the control for significance analysis. Data are presented as mean±SD (n=3). $^{*}P < 0.05$, $^{**}P < 0.01$, $^{***}P < 0.001$.

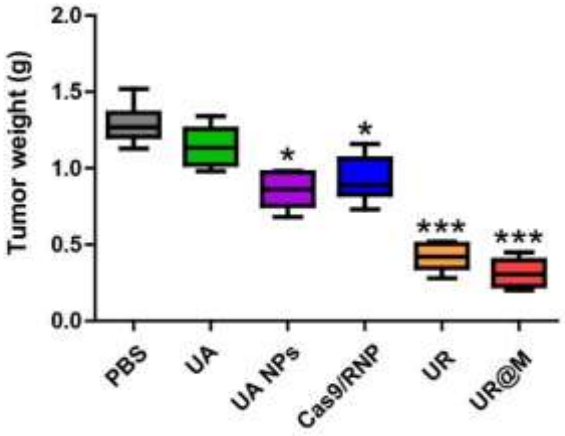

Figure S16. The weight of excised tumor was detected after the *in vivo* experiment. PBS group was treated as the control for significance analysis. Data are presented as mean±SD (n=3). $^{*}P < 0.05$, $^{**}P < 0.01$, $^{***}P < 0.001$.

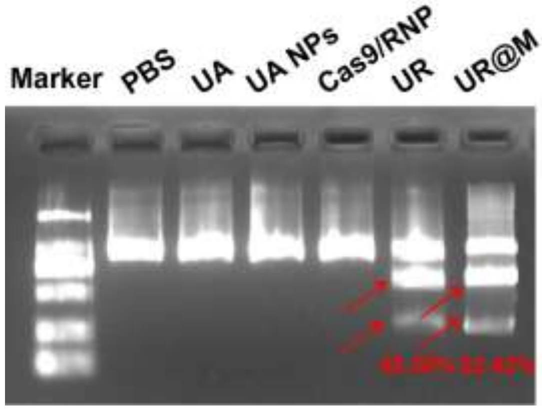

Figure S17. The evaluation of the gene editing of PD-L1 in the tumors after the treatment of UR and UR@M by T7E1 assay.

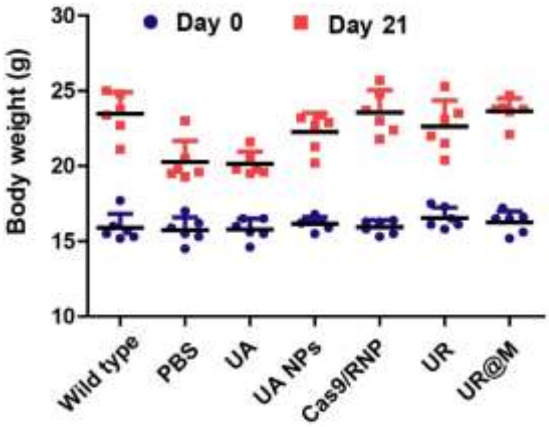

Figure S18. Body weight of tumor-bearing mice post the indicated treatment during the 21 days (*n*=6).

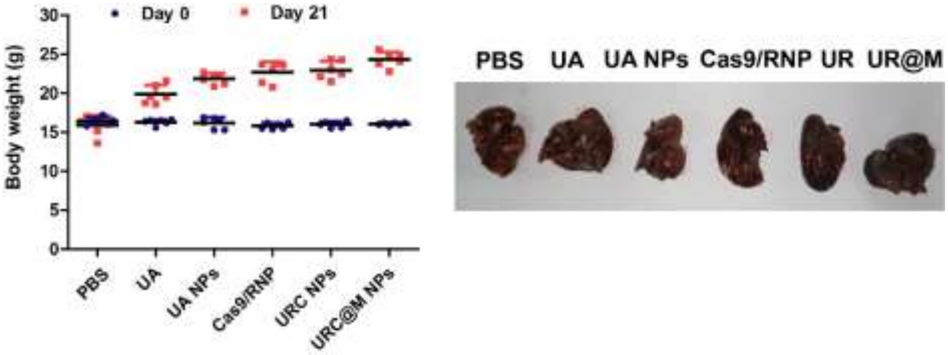

Figure S19. The weight of HCC orthotopic tumor mice and the pictures of liver after the treatment.

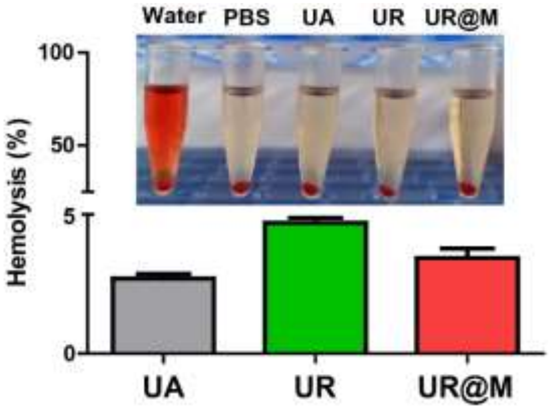

Figure S20. Hemolytic test of UA NPs, UR NPs, and UR@M NPs. The water and PBS were used as the positive (hemolysis ratio: 100 %) and negative (hemolysis ratio: 0 %) control. It was considered a hemolysis effect when the hemolysis ratio was more than 5 %. Data are presented as mean±SD (n=3).

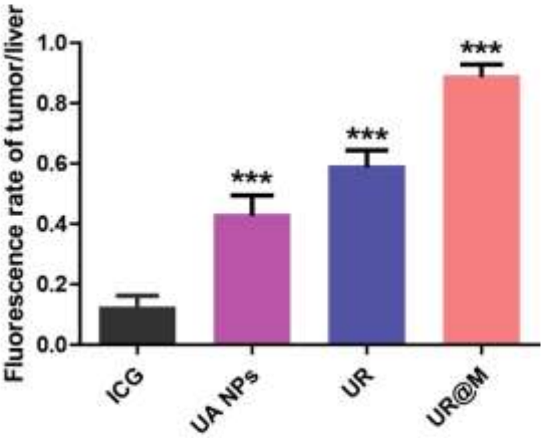

Figure S21. The fluorescence ratio of tumor/liver ratio of UR NPs, UR NPs and UR@M NPs with the ICG group as the control. Data are presented as mean±SD (n=3). $^{***}P < 0.001$.

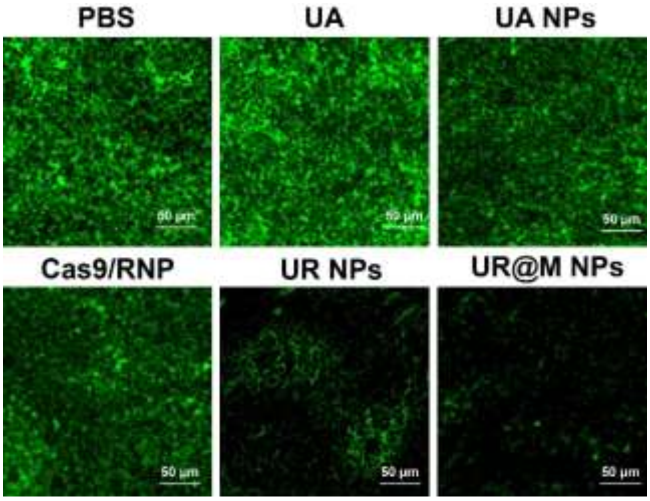

Figure S22. The PD-L1 expression on tumors following the *in vivo* treatment was examined by immunofluorescent staining. Scale bar= 50 μm.

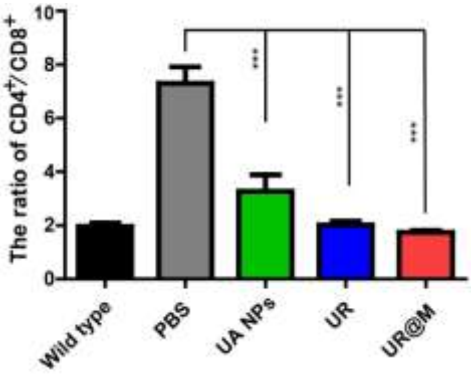

Figure S23. The ratio of $CD4^+$ T cells to $CD8^+$ T cells in the blood. The normal physiological environment ratio was about 1.4-2.0. PBS group was treated as the control for significance analysis. Data are presented as mean±SD (*n*=3). $^{***}P < 0.001$.

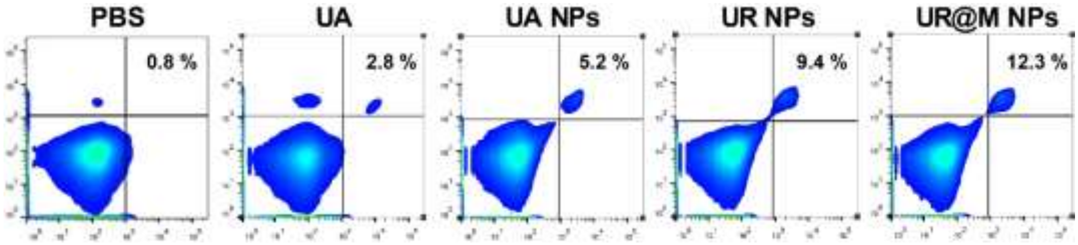

Figure S24. Evaluation of tumor immune infiltration after the *in vivo* treatment. The percentage of CTLs was examined in tumor tissues by flow cytometry.
